# Study Protocol: A randomized controlled trial evaluating the effect of family-based behavioral treatment of childhood and adolescent obesity–The FABO-study

**DOI:** 10.1186/s12889-016-3755-9

**Published:** 2016-10-21

**Authors:** Hanna F. Skjåkødegård, Yngvild S. Danielsen, Mette Morken, Sara-Rebekka F. Linde, Rachel P. Kolko, Katherine N. Balantekin, Denise E. Wilfley, Pétur B. Júlíusson

**Affiliations:** 1 Department of Medicine, the Obesity Outpatient Clinic, Haukeland University Hospital, N-5021 Bergen, Norway; 2 Department of Clinical Psychology, University of Bergen, Bergen, Norway; 3 Department of Psychiatry, University of Pittsburgh School of Medicine, Pittsburgh, PA USA; 4 Department of Psychiatry, Washington University School of Medicine, St. Louis, MO USA; 5 Department of Medicine, Washington University School of Medicine, St. Louis, MO USA; 6 Department of Pediatrics, Washington University School of Medicine, St. Louis, MO USA; 7 Department of Psychology, Washington University School of Medicine, St. Louis, MO USA; 8 Department of Clinical Science, University of Bergen, Bergen, Norway; 9 Department of Physiotherapy, Haukeland University Hospital, Bergen, Norway

**Keywords:** Childhood obesity, Randomized controlled trial, Family-based behavioral treatment

## Abstract

**Background:**

The purpose of the FABO-study is to evaluate the effect of family-based behavioral social facilitation treatment (FBSFT), designed to target children’s family and social support networks to enhance weight loss outcomes, compared to the standard treatment (treatment as usual, TAU) given to children and adolescents with obesity in a routine clinical practice.

**Methods:**

Randomized controlled trial (RCT), in which families (*n* = 120) are recruited from the children and adolescents (ages 6–18 years) referred to the Obesity Outpatient Clinic (OOC), Haukeland University Hospital, Norway. Criteria for admission to the OOC are BMI above the International Obesity Task Force (IOTF) cut-off ≥ 35, or IOTF ≥ 30 with obesity related co-morbidity. Families are randomized to receive FBSFT immediately or following one year of TAU. All participants receive a multidisciplinary assessment. For TAU this assessment results in a plan and a contract for chancing specific lifestyle behaviors. Thereafter each family participates in monthly counselling sessions with their primary health care nurse to work on implementing these goals, including measuring their weight change, and also meet every third month for sessions at the OOC. In FBSFT, following assessment, families participate in 17 weekly sessions at the OOC, in which each family works on changing lifestyle behaviors using a structured cognitive-behavioral, socio-ecological approach targeting both parents and children with strategies for behavioral maintenance and sustainable weight change.

Outcome variables include body mass index (BMI; kg/m^2^), BMI standard deviation score (SDS) and percentage above the IOTF definition of overweight, waist-circumference, body composition (bioelectric impedance (BIA) and dual-X-ray-absorptiometry (DXA)), blood tests, blood pressure, activity/inactivity and sleep pattern (measured by accelerometer), as well as questionnaires measuring depression, general psychological symptomatology, self-esteem, disturbed eating and eating disorder symptoms. Finally, barriers to treatment and parenting styles are measured via questionnaires.

**Discussion:**

This is the first systematic application of FBSFT in the treatment of obesity among youth in Norway. The study gives an opportunity to evaluate the effect of FBSFT implemented in routine clinical practice across a range of youth with severe obesity.

**Trial registration:**

ClinicalTrails.gov NCT02687516. Registered 16th of February, 2016

**Electronic supplementary material:**

The online version of this article (doi:10.1186/s12889-016-3755-9) contains supplementary material, which is available to authorized users.

## Background

There has been a global increase in the prevalence and severity of overweight and obesity amongst both adults and children [1, 2]. The World Health Organization (WHO) states that childhood obesity is one of the most serious public health challenges of the 21st century [2].

Childhood obesity is associated with a wide array of negative health consequences such as psychosocial problems, obstructive sleep apnea, increased cardio-metabolic risk and Type 2 Diabetes [3–5], resulting in considerable burden for the individual child as well as socio-economic consequences [6, 7]. In addition, childhood obesity has great impact on adulthood, and is associated with adult obesity [8] and all-cause mortality in adulthood [9]. A recent study [9] with 2.3 million participants between the ages of 16 and 19 years found that a graded increased risk for cardiovascular and all-cause mortality during 40 years of follow-up started among participants with a body mass index (BMI; kg/m^2^) currently accepted within the normal range (from 50th to 74th percentiles).

The need for developing potent treatments for childhood obesity has been recognized for decades and a substantial body of treatment research exists [10–12]. There is also substantial evidence indicating that it is difficult to get more than modest weight loss when treating adolescents with severe obesity [13, 14].

Several reviews and meta-analyses have summarized the results of different childhood obesity treatments and provided recommendations as to which treatments are the most efficacious and which treatment components are predictive of favorable treatment outcomes [11, 12, 15, 16]. Specifically, lifestyle interventions that teach behavioral techniques that focus on incorporating the behavior changes into daily life routines, and are delivered in a family-based format (i.e., targeting both the child and parent), seem to be the most efficacious treatment for childhood obesity [11, 15–18]. However, it is important to note that much of this research has been conducted within efficacy trials. Thus, effectiveness trials, which focus on the applicability and validity of the treatment in usual health care settings with a less selected group of participants, are warranted, as few have been conducted [19].

Childhood obesity treatment of medium-to high intensity and longer duration have been found to be most efficacious [20]. Best effect for treatments that included more than 26 sessions are found in one review [20]. There is however some discussion about how prolonged the treatment needs to be to yield meaningful weight loss outcomes. For instance, one review suggests that interventions that last about four months are as efficacious as treatments of longer duration [21]. A meta-analyses of intervention studies from 2002–2015 [18] finds that weight loss treatment for children has evolved over the past decade, especially in that recent studies report longer duration and follow-up than in the 1990s [18]. Despite these findings that indicate the importance of higher-intensity treatment, many national guidelines for addressing childhood obesity recommend weight surveillance and brief lifestyle counseling in primary care service for children with obesity and their families [22]. However, this approach is not effective, at least not for children and adolescents with severe obesity [22, 23].

Recently, there has been consensus that obesity is a disease [24]. Classifying obesity as a chronic health condition increases the understanding of the fact that extended follow-up after an intensive treatment phase is necessary for weight loss maintenance. Long-term maintenance of treatment effects is a challenge with obesity treatment across both adults and children [25–27]. Family-based behavioral treatments for childhood obesity have, importantly, demonstrated promising long-term effects compared to other treatments [15, 28]. A ten-year follow-up study found that 34 % of the participants who entered the treatment program at age 6–12 had reduced their percent overweight by at least 20 %, and 30 % had BMIs that were no longer in the obese range [28].

Dropout is a substantial concern in treatment of both adult and pediatric obesity [11, 29, 30], but only a limited number of studies have examined the predictors for dropout [29, 31, 32]. Studies show mixed results regarding barriers to treatment participation. Some studies have found higher number of barriers in families who end the treatment program prematurely [32, 33], while another found few differences in degree of barriers among treatment completers compared to non-completers [31]. Post-treatment reported barriers in prior studies include high degree of family stressors, parent-adolescent conflict, lack of time and interest, interference with school schedules, disappointment with amount of weight-loss, and treatment taking place too far from home [31, 32, 34]. Further research on predictors for dropout and barriers to treatment participation, both among non-completers and completers, has important implications for clinical practice [30].

Another subject of clinical importance is to identify pre-treatment factors related to poorer treatment response. Together with knowledge about predictors for dropout and barriers to treatment participation, these factors can identify at risk groups and tailor childhood obesity treatment to increase treatment response. Several pre-treatment factors have been associated with weight loss outcomes in previous research on childhood obesity treatment [30, 31, 35–38], including child age, gender, child initial weight status [36, 38], child mental health problems [36, 37], and parents’ degree of motivation [30, 31, 35, 36]. However, there seems to be a lack of studies investigating the influence of family variables and broader social network support as predictors of treatment response.

The aim of this paper is to describe and explain the design and evaluation of the Family-based behavioral treatment of childhood obesity (FABO)−study targeting children and adolescents with obesity and their families. The description of the study protocol follows the checklist of the CONSORT statement for randomized trials [39] (Additional files 1 and 2).

## Methods/Design

### Intervention

Family-based behavioral social facilitation treatment (FBSFT) is founded on the principles of standard family-based behavioral treatment for obesity, which is the most well documented approach for childhood obesity [11].

FBSFT has an intensive treatment phase including weekly family meetings over 17 consecutive weeks with the same health care worker at the Obesity Outpatient Clinic (OOC), Haukeland University Hospital, Norway. All the health care workers on the multidisciplinary treatment team at the OOC are trained in FBSFT prior to treatment delivery. The multidisciplinary team consists of a pediatrician, nutritionist, physiotherapist, nurse and psychologist. Throughout the study period, the treatment team will have weekly meetings to discuss the patients in addition to monthly supervision sessions with the research team in St. Louis, MO, USA and Pittsburgh, PA, USA through video-conferences.

There are session-specific components and goals for each of the 17 sessions (see Table 1 for complete outline). The treatment targets healthy lifestyle changes in both the children and parents in the areas of diet, physical activity, sedentary activity, sleep and social function. The dietary and physical activity guidance used in the study is based on the Traffic Light Diet [40] in which foods and activities are organized into green, yellow and red groups. Green meaning “go,” yellow meaning “sometimes” and red meaning “limit.” The treatment focuses on implementing the behavior change across all the different settings in the family members’ daily lives (i.e., within the home/family environment, peer network environment, community environment).

**Table 1:** Session topics in family-based behavioral facilitation treatment (FBSFT)

Phase	Session	FBSFT Topic
1. Individual and Home Contex	1-2	Introduction to the treatment; plan for the Traffic Light Diet; personalized treatment plan
	3	Healthy and regular eating, communicating with the family about lifestyle changes
	4	Sedentary activity; sleep routines
	5	Physical activity; lifestyle activity
	6	Creating a healthy family and home environment; problem solving skills
	7	Healthy self-instructions; emotions/stress and eating behavior
2. Peer Contex	8	Peers as a support for healthy behaviors (arranging healthy activities with others); assessment of social network
	9	High risk situations (parties, holidays and vacations); prompts for eating and physical activity
	10	A healthy peer environment; communicating with peers about new and healthy habits
	11	Taking on Teasing
3. Community Contex	12	Physical activity and the assessment of RED food in the environment/neighborhood
	13	To be active in your neighborhood; join groups or teams; to elicit support for healthy habits in your neighborhood/environment
	14	To fight weight stigmatization; influences from the media; to build a positive self-image and body image
	15	High risk situations (restaurants and fast food); to focus on healthy habits at school and work
4. Cross-contextual	16	To plan for healthy habits; relapse prevention and consolidating skills across different contexts
	17	Reviewing goals and skills; ending well; planning ahead

Through the treatment sessions, the families are taught a set of behavioral and cognitive techniques for promoting healthy behavior change and dealing with mechanisms that maintain unhealthy lifestyle behaviors:

- Self-monitoring. Both the parents and children monitor their eating, weight and activity on a week-to-week basic going through the intensive treatment phase
- Goal setting, planning and reward systems
- Stimulus control
- Emotion regulation strategies
- Reframing negative automatic thoughts
- Communication and interpersonal skills
- Parenting strategies
- Healthy modeling from the parents
- Healthy methods of self-evaluation and self-assertion

After the intensive treatment phase, the families receive monthly follow-up treatment for 18 months through collaboration between specialty and primary care: monthly follow-up sessions with their school nurse (primary care) and follow-up sessions every third month at the OOC. The focus for these sessions is maintenance of healthy habits.

For families assigned to standard treatment (treatment as usual, TAU), the treatment consists of a post-assessment meeting between a health care worker at the OOC and the family, agreeing on behavioral goals for changing lifestyle, a plan for the implementation of new behaviors and goals for weight loss. Each family also participates in monthly counselling session with their local health care nurse to work on implementing these goals, including measuring their weight change, and also meets every third month for sessions at the OOC for assessments, evaluation of progress and revision of goals. TAU is delivered over the course of 12 months.

### Study objectives and hypotheses

The objectives and hypotheses of the current study are:

- To evaluate the effect of FBSFT compared to TAU for severe childhood and adolescent obesity in a common health care setting. The primary outcome is weight status, assessed as BMI, BMI standard deviation scores (SDSs) and percentages above the IOTF cut-off for overweight (%IOTF-25) [41]. Secondary outcomes are other weight-related anthropometric measurements (waist circumference (WC) and Waist-to-height ratio (WHtR) and corresponding SDSs), body composition (BIA, DXA), blood test, blood pressure, eating habits, sleep, physical activity as well as psychological well-being and parenting style.
  - ○ We hypothesized that FBSFT will be superior to TAU in improving these parameters both during the intensive treatment period and during the follow-up period.
- To identify predictors of treatment success and treatment drop out with a focus on family variables, socioeconomic status, social network and mental health.
  - ○ We expect children from families in which both parents participate in treatment to demonstrate better weight loss outcome and have lower dropout rate.
  - ○ We expect children of parents living together to demonstrate better treatment effects and have lower dropout rate.
  - ○ We expect that parental (self-reported) weight status will influence treatment effects and dropout rate; higher BMI category will predict poorer treatment effects and higher dropout rate.
  - ○ We expect that lower socio-economic status and limited social network will predict poorer treatment effects and higher dropout rate.
  - ○ We expect poorer treatment effect and higher dropout rate among participants with psychological comorbidities.
- To evaluate experienced barriers to treatment, and how these factors influence children’s and parents treatment response. Barriers to treatment are classified into four groups: competing activities/life stressors, relevance of treatment, treatment issues (logistics) and critical events.
  - ○ We expect lower degree of competing activities/life stressors to predict better treatment response and lower dropout rate.
  - ○ We expect higher degree of experienced treatment relevance to predict better treatment response and lower dropout rate.
  - ○ We expect lower degree of treatment issues and lower number of critical events to predict better treatment response and lower dropout rate.
- To evaluate FBSFT implementation and acceptability for children, parents and health care workers. This is an exploratory study using a brief interview to investigate parents’, children’s and health care workers’ experiences with the treatment.

### Trial design

**Fig. 1:**
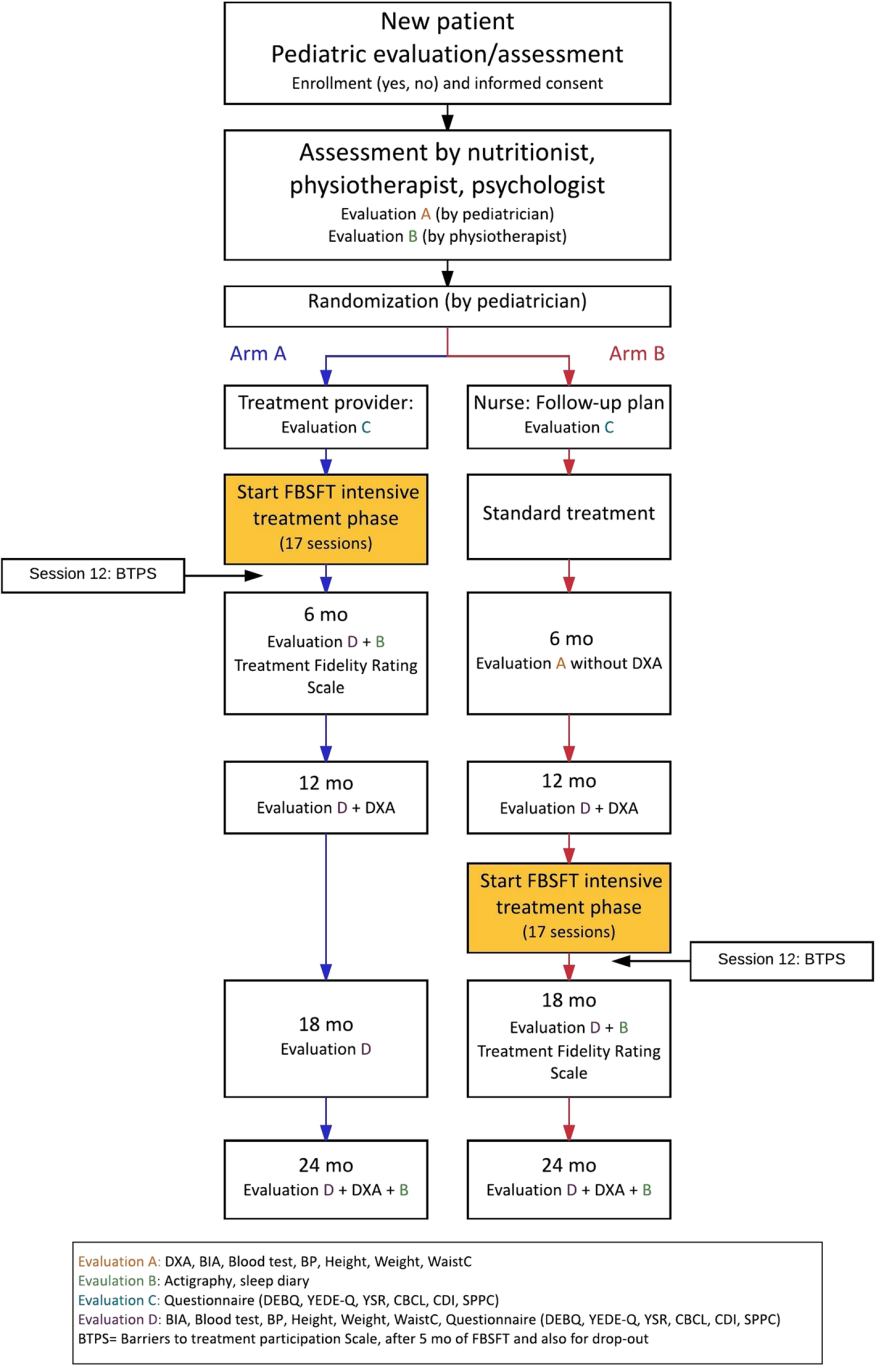
Flow sheet for the FABO-study

The FABO-study is a randomized clinical trial (RCT) using a wait-list control design in which all recruited families of children with severe obesity will receive FBSFT at some point. All families go through initial assessments at the Obesity Outpatient Clinic (OOC) at Haukeland University Hospital, and can choose to give informed consent to participate. If informed consent is given, the families are randomized to either FBSFT (arm A) or TAU followed by FBSFT one year later (arm B). The overall study design is summarized in Fig. 1. Flow sheet for the FABO-study.

### Blinding

The data manager and statisticians are blinded to group allocation until analyses are conducted. Blinding of participants and/or the treatment team was not possible due to the nature of the study.

### Inclusion criteria

The sample will consist of 120 children and adolescents (aged 6–18 years) referred to the OOC by their general practitioner. Criteria for admission to the clinic is an International Obesity Task Force (IOTF) BMI ≥ 35, or ≥ 30 [41] with obesity related co-morbidity. The child/adolescent participates in the treatment together with her/his family, such that both the child and a least one of the parents agree to actively participate in the treatment.

### Exclusion criteria

Families are excluded if either the children or parents experience severe somatic or psychiatric illness that affect weight or adherence to the treatment program, or current participation in other obesity treatment programs.

### Strategies to improve adherence to intervention protocol

A standardized checklist for data collection for each child/adolescent participating in the study is created after randomization. Once per month the checklists are reviewed, and participants with missing data are reminded by either text message or phone call to complete the assessments. In the intensive treatment phase of FBSFT, sessions are rescheduled so that each family receives the same content, while delivering the sessions within a 6 month period. Sessions are rescheduled within the week when possible, and are combined with the next session or delivered by phone if the family is unable to attend (e.g., due to weather or driving conditions, scheduling conflicts). Health care workers meet weekly for on-site supervision of intervention delivery, and monthly for Skype supervision with the research team in St.Louis, MO, USA and Pittsburgh, PA, USA.

### Sample size

One hundred twenty families will be recruited, with 60 families in each treatment arm. Power estimates were calculated using G*Power, version 3.1.3. [42, 43] With two treatment groups (TAU, FBSFT), 3 measurement points (pre-treatment, 6 months post-treatment and 12 month post-treatment) and a correlation of 0.5 between the measurement points, alpha .05, power .80, a small (*f* = 0.10), moderate (*f* = 0, 25) and high (*f* = 0.40) effect size would demand respectively sample sizes of 164, 28 and 12 participants to detect a significant group X time interaction on the primary outcome. For regression analysis of predictors of treatment outcome, a small to moderate effect size is expected (*f* = 0.15), and when testing the increase in R^2^ by adding 1 predictor to an analysis including 5 predictors in total, a sample size of 55 persons will be needed.

### Randomization

After informed consent is given and the initial assessment is completed, each participant is randomly allocated to either receive FBSFT immediately (arm A) or following one year of TAU (arm B). The randomization is performed by the pediatrician and allocation to the two groups is done by extracting a random, sealed envelope from a sealed folder. At the beginning of the study, there were 120 sealed envelopes: 60 with the letter A (for arm A) and 60 with the letter B (for arm B).

## Outcome measures

Measurement points are pre-treatment, post-treatment (i.e., 6 months from pre-treatment), at 12 months, 18 months and 24 months. See Fig. 1 for overview.

### Anthropometrical measures

Trained assessors at the OOC will measure height and weight for calculation of BMI (kg/m2), WC and body composition (measured with bio impedance (BIA), InBody 720). BMI will be converted to SDS using the extended IOTF [41, 44] BMI references. The percentage of the IOTF 25 threshold (%IOTF-25) is calculated as 100*(BMI/IOTF 25), where BMI is the child’s weight divided by height squared (kg/m^2^), and IOTF 25 is the BMI that corresponds to the IOTF threshold for overweight for the child’s age and sex. Dual-energy X-ray absorptiometry (DXA)-scans for determining the distribution of fat and muscle tissue will be conducted at the Department of Rheumatology at Haukeland University Hospital.

### Physiological measures

Blood samples will be drawn in the morning after an overnight fast and include measurements of total-cholesterol, high density lipoprotein (HDL), low-density lipoprotein (LDL), triglycerides (TG), aspartate transaminase (ASAT), alanine transaminase (ALAT), creatinine, glycated hemoglobin (HbA1c) and fasting insulin, c-peptide and glucose, thyroid stimulating hormone (THS), free thyroxine (fT4), and C-reactive protein (CRP). A bio-bank for the storing and registering of biological materials has been approved. Trained assessors at the OOC will measure blood pressure.

### Physical activity and sleep patterns

Physical activity and sleep patterns will be measured using Actiwatch 2 (Phillips). The actiwatch devices are wrist-worn accelerometers that records all uni-axial movement over 0.05G in thirty-second epochs. The actiwatch will be worn on the non-dominant wrist for seven consecutive days. A wrist-worn accelerometer was chosen over hip-worn accelerometers to ensure compliance [45]. Wrist-worn accelerometers are validated for use both as a measure of physical activity, inactivity and sleep and are recommended for use in studies evaluating lifestyle interventions for obesity among children and adults [46–48].

### Psychological measures

The following five questionnaires will be used in the study:

*The Child Behavior Checklist (CBCL)* [49], a 138-item scale assessing behavioral and emotional symptoms in children that has both a child/youth and parent form. Several studies have provided evidence of the instruments psychometric properties and stability [49–51].

*Children’s Depression Inventory (CDI)* [52], a 27-item self-report measure assessing the cognitive, affective and behavioral symptoms of depression in children (7–17 years). The psychometric properties of the scale have generally been found to be acceptable [53, 54].

*Self-Perception Profile for Children (SPPC)* [55], a self-report measure of self-perception or self-esteem in children aged 8 to 14 years widely used for research purposes. The questionnaire includes 36 statements and the children are asked to evaluate to which degree the statement fits their thoughts about themselves. The internal reliability has been demonstrated to be high [56].

*The Dutch Eating Behavior Questionnaire Child version (DEBQ)* [57], a measure of disordered eating behaviors in children and youth. DEBQ consists of three sub-scales: emotional eating, external eating, and restrained eating. The questionnaire is increasingly used for research on youth with overweight and psychometric properties have in general been found acceptable [58, 59].

*The Youth Eating Disorder Examination-Questionnaire (YEDE-Q)* [60], a self-reported measure of eating patterns and eating disorder psychopathology. The YEDE-Q is a self-report version of the Child Eating Disorder Examination (ChEDE) and was designed to include measurements of binge eating in youth [61]. The YEDE-Q has been validated using the ChEDE as an assessment of eating-related pathology in overweight youth [61].

### Other measurements

*Barriers to treatment participation scale (BTPS)* [62], a 44-item scale developed and validated to address drop-out from treatment with out-patient psychological treatment of children and adolescents. The scale is found to yield high levels of internal consistency and to be predictive of treatment dropout and weeks spent in treatment [62, 63].

*The Parenting Scale (PS)* [64], 30-item questionnaire designed to measure different parental disciplines with children and youth. The scale is widely used for research and clinical purposes. The internal consistency and test-retest stability have been found to be acceptable to good, and the validity of the instrument has been demonstrated in several studies [65, 66].

## Planned data analysis

A two-way MANOVA with one repeated-measure factor (Time: pre-intervention/post-intervention), and one between-group factor (Treatment Group: FBSFT vs. TAU) will be conducted to assess the impact of treatment on primary and secondary outcomes.

A one way within-subjects MANOVA will be used to analyze the effect of time (pre-treatment, post-treatment, 12-, 18-, 24 months post treatment) on primary and secondary outcomes.

In order to identify factors associated with treatment success and treatment drop out, multiple regression analyses will be conducted. The dependent variables are %IOTF-25 and drop-out status (yes/no). The predictors are: both parents participating in treatment, parental marital status, self-reported parental weight, socio-economic status and social network, psychological comorbidities.

In order to identify barriers to treatment and how these barriers influence children’s and parents’ treatment response, multiple regression analyses will be conducted. The dependent variable is %IOTF-25. The predictors are: competing activities/life stressors, relevance of treatment, treatment issues (logistics) and critical events.

Intention-to-treat analyses will be conducted and effect sizes calculated for treatment effects.

### Time plan for the FABO-study

In this study, we aim to recruit 120 families of children with severe obesity. Enrollment of families to the study began in January 2014 and we anticipate that recruitment will be completed by Autumn 2017.

## Discussion

To our knowledge, this study is the first RCT conducted in Scandinavia evaluating the effect of family-based behavioral treatment of childhood and adolescent obesity delivered in a chronic care model that comprises specialty care and routine clinical practice. The 2011 guidelines from the Norwegian Directorate of Health concerning treatment of overweight children [67], recommend the use of more structured family-based behavioral treatments for severely obese children. However, the availability of such programs has been limited, as well as the possibilities to get training in delivering this type of treatment. This study offers an opportunity to evaluate the effect of this treatment in routine clinical practice. After the study period, FBSFT is likely to be considered as a standard option for treatment, then as a part of stepped care treatment, meaning that non-responders to standard care will advance to this more intensive and targeted treatment.

## Additional files

Additional file 1: SPIRIT 2013 Checklist. Completed checklist for the study protocol article and related document. (DOC 121 kb)
Additional file 2: Extended information. Study Protocol: A randomized controlled trial evaluating the effect of family-based behavioral treatment of childhood and adolescent obesity – The FABO-study. Description of data: Administrative information requested by SPIRIT 2013 Checklist that are not addressed in the study protocol article. (DOCX 19 kb)

## Abbreviations

BIA: Bioelectric impedance
BMI: Body mass index
BTPS: Barriers to treatment participation scale
CBCL: The Child Behavior Checklist
CDI: Children’s Depression Inventory
ChEDE: Child Eating Disorder Examination
DEBQ: The Dutch Eating Behavior Questionnaire Child version
DXA: Dual-X-ray-absorptiometry
FBSFT: Family-based behavioral social facilitation treatment
IOTF: International Obesity Task Force
OOC: The Obesity Outpatient Clinic
PS: The Parenting Scale
RCT: Randomized controlled trial
SPPC: Self-Perception Profile for Children
TAU: Treatment as usual
WC: Waist circumference
WHO: The World Health Organization
WHtr: Waist-to-height ratio
YEDE-Q: The Youth Eating Disorder Examination-Questionnaire
