# Supplementary material for: Study Protocol: A randomized controlled trial evaluating the effect of family-based behavioral treatment of childhood and adolescent obesity–The FABO-study
Source: BMC Public Health. 2016 Oct 21;16:1106. doi: 10.1186/s12889-016-3755-9 (PMC5073413; doi:10.1186/s12889-016-3755-9)
Supplement: Additional file 2: — Extended information. Study Protocol: A randomized controlled trial evaluating the effect of family-based behavioral treatment of childhood and adolescent obesity – The FABO-study. Description of data: Administrative information requested by SPIRIT 2013 Checklist that are not addressed in the study protocol article. (DOCX 19 kb) [file 12889_2016_3755_MOESM2_ESM.docx]

**Extended information. Study Protocol: A randomized controlled trial evaluating the effect of family-based behavioral treatment of childhood and adolescent obesity – The FABO-study**

Trial registration

**Figure 1.WHO Trial Registration Data Set**

| **Data category** | **Information** |
| --- | --- |
| Primary registry and trial identifying number | ClinicalTrails.gov NCT02687516 |
| Date of registration in primary registry | 16 February, 2016 |
| Secondary identifying numbers | Regional Committee for medical and health research ethics (REK) – Western Norway. REK number: 2013/1300 |
| Source(s) of monetary or material support | Research grants from the Western Norway Regional Health Authority |
| Primary sponsor | No sponsor |
| Secondary sponsor(s) | No sponsor |
| Contact for public queries | YD ([yngvild.danielsen@uib.no](mailto:yngvild.danielsen@uib.no)) and PBJ ([petur.juliusson@uib.no](mailto:petur.juliusson@uib.no)) |
| Contact for scientific queries | YD ([yngvild.danielsen@uib.no](mailto:yngvild.danielsen@uib.no)) and PBJ ([petur.juliusson@uib.no](mailto:petur.juliusson@uib.no)) |
| Public title | Study Protocol: A randomized controlled trial evaluating the effect of family-based behavioral treatment of childhood and adolescent obesity – The FABO-study |
| Scientific title | Study Protocol: A randomized controlled trial evaluating the effect of family-based behavioral treatment of childhood and adolescent obesity – The FABO-study |
| Countries of recruitment | Norway |
| Health condition(s) or problem(s) studied | Childhood and adolescent obesity |
| Intervention(s) | Family-based behavioral social facilitation treatment (FBSFT) of obesity compared to standard treatment given to children and adolescents with obesity in a routine clinical practice |
| Key inclusion and exclusion criteria | Ages eligible for study: 6-18 years |
|  | Inclusion criteria: IOTF BMI ≥ 35 or ≥ 30 with obesity related co-morbidity. At least one of the parents agrees to participate in the treatment together with the child/adolescent |
|  | Exclusion criteria: Severe somatic or psychiatric illness, in children/adolescent or parents, that affect weight or adherence to the treatment program. Current participation in other obesity treatment programs |
| Study type | Interventional |
|  | Allocation: Randomized controlled trial |
|  | Primary purpose: Treatment |
| Date of firs enrolment | February 2014 |
| Target sample size | 120 |
| Recruitment status | Recruiting |
| Primary outcome(s) | Weight status, assessed as BMI (kg/m^2^), BMI standard deviation scores (SDS) and percentages above the IOTF cut-off for overweight (%IOTF-25) |
| Key secondary outcomes | Other weight-related anthropometric measurements (waist circumference (WC) and Waist-to-height ratio (WHtR) and corresponding SDSs), body composition (BIA, DXA), blood test, blood pressure, eating habits, sleep, physical activity as well as psychological well-being and parenting style, drop-out status (yes/no) and number of treatment sessions attended |

Data management and confidentiality

Data from enrolled participants are collected both on paper forms and electronic. Identifying information will be depersonalized, and the linking codes are kept looked down behind two looked doors in a looked filling cabinet. The data collected on paper forms will be kept looked down in a looked filling cabinet behind two looked doors. The filing cabinet with the identifying information and the filling cabinet with collected data are kept in two separate offices. Electronic collected data will be kept in encrypted digital files within password-protected folders at the local research server at Haukeland University Hospital, Norway. The access to both the data collected on paper and the electronic data are restricted to HFS, YD and PBJ.

Data monitoring

There will not be a data monitoring committee (DMC) in the FABO-study. A DMC is considered unnecessary, as the study is a minimal risk study.

Protocol amendments

If important protocol modifications are necessary, a letter with notification about the changes will be submitted to the Regional Committee for medical and health research ethics (REK) – Western Norway.

Access to data

Access to the final trial dataset are given to YSD, RK, DW and PBJ. Master`s degree- and PHD-students will be granted access to coded, depersonalized datasets after approval from YSD and PBJ.

Dissemination policy

The results of the study will be published in international peer-reviewed journals, and presented in relevant international congresses.

Biological specimens

A local research BIOBANK at Haukeland University Hospital, Norway is affiliated with the study. The depersonalized samples will be kept in the BIOBANK and used for analysis affiliated with the FABO- study.
